# Vitamin E Isoforms Differentially Regulate Intercellular Adhesion Molecule-1 Activation of PKCα in Human Microvascular Endothelial Cells

**DOI:** 10.1371/journal.pone.0041054

**Published:** 2012-07-17

**Authors:** Hiam Abdala-Valencia, Sergejs Berdnikovs, Joan M. Cook-Mills

**Affiliations:** Allergy-Immunology Division, Northwestern University Feinberg School of Medicine, Chicago, Illinois, United States of America; Hungarian Academy of Sciences, Hungary

## Abstract

**Aims:**

ICAM-1-dependent leukocyte recruitment *in vivo* is inhibited by the vitamin E isoform d-α-tocopherol and elevated by d-γ-tocopherol. ICAM-1 is reported to activate endothelial cell signals including protein kinase C (PKC), but the PKC isoform and the mechanism for ICAM-1 activation of PKC are not known. It is also not known whether ICAM-1 signaling in endothelial cells is regulated by tocopherol isoforms. We hypothesized that d-α-tocopherol and d-γ-tocopherol differentially regulate ICAM-1 activation of endothelial cell PKC.

**Results:**

ICAM-1 crosslinking activated the PKC isoform PKCα but not PKCβ in TNFα-pretreated human microvascular endothelial cells. ICAM-1 activation of PKCα was blocked by the PLC inhibitor U73122, ERK1/2 inhibitor PD98059, and xanthine oxidase inhibitor allopurinol. ERK1/2 activation was blocked by inhibition of XO and PLC but not by inhibition of PKCα, indicating that ERK1/2 is downstream of XO and upstream of PKCα during ICAM-1 signaling. During ICAM-1 activation of PKCα, the XO-generated ROS did not oxidize PKCα. Interestingly, d-α-tocopherol inhibited ICAM-1 activation of PKCα but not the upstream signal ERK1/2. The d-α-tocopherol inhibition of PKCα was ablated by the addition of d-γ-tocopherol.

**Conclusions:**

Crosslinking ICAM-1 stimulated XO/ROS which activated ERK1/2 that then activated PKCα. ICAM-1 activation of PKCα was inhibited by d-α-tocopherol and this inhibition was ablated by the addition of d-γ-tocopherol. These tocopherols regulated ICAM-1 activation of PKCα without altering the upstream signal ERK1/2. Thus, we identified a mechanism for ICAM-1 activation of PKC and determined that d-α-tocopherol and d-γ-tocopherol have opposing regulatory functions for ICAM-1-activated PKCα in endothelial cells.

## Introduction

Leukocytes bind to endothelial cell adhesion molecules such as intercellular adhesion molecule-1 (ICAM-1) and vascular adhesion molecule-1 (VCAM-1) during their migration across endothelial barriers at sites of inflammation. In allergic inflammation, recruitment of lymphocytes and eosinophils is dependent on binding to ICAM-1 and VCAM-1 as demonstrated by in vivo administration of blocking antibodies for these adhesion molecules or ICAM-1 knockout mice [1], [2], [3], [4], [5]. We have reported that, in allergic lung inflammation in mice, the recruitment of lymphocytes and eosinophils is inhibited by the vitamin E isoform d-α-tocopherol and elevated by the vitamin E isoform d-γ-tocopherol [6]. These two isoforms of tocopherols differ by one methyl group [7], [8]. The tocopherol isoform-specific regulation of leukocyte recruitment to the lung in allergic responses occurs without altering expression of several mediators of inflammation including cytokines, chemokines and VCAM-1 [6]. Interestingly, leukocyte migration across endothelium expressing VCAM-1 is regulated by d-α-tocopherol and d-γ-tocopherol when endothelial cells are pretreated with tocopherols but not when leukocytes are pretreated with tocopherols [6]. Pretreatment of endothelial cells with d-α-tocopherol inhibits leukocyte transendothelial migration whereas pretreatment of endothelial cells with d-γ-tocopherol elevates migration [6]. Furthermore, d-α-tocopherol inhibits and d-γ-tocopherol elevates VCAM-1 signaling in endothelial cells. However, it is not known whether these tocopherol isoforms, which regulate ICAM-1-dependent leukocyte recruitment in vivo, also differentially regulate ICAM-1 signaling in endothelial cells.

It is reported that the cytoplasmic domain of ICAM-1 is required for leukocyte transmigration [9], [10], [11], suggesting that ICAM-1 signals are necessary for leukocyte transendothelial migration on ICAM-1. It is reported that binding to ICAM-1 activates several signals in endothelial cells. Engagement of endothelial ICAM-1 by leukocytes, antibodies or fibrinogen induces an increase in endothelial cell intracellular calcium [12], [13], [14], [15], cytoskeletal changes [16], [17], and xanthine oxidase (XO)-dependent generation of reactive oxygen species (ROS) [18], [19], [20]. ICAM-1-dependent ROS production stimulates phosphorylation of p38 kinase and cytoskeleton-associated proteins [17], [21], [22], [23], [24]. Binding to ICAM-1 also activates a calcium/PLCγ_1_/PKC pathway for the stimulation of Src phosphorylation of cytoskeletal proteins during leukocyte migration across brain endothelial cell lines [24]. Chelating calcium or inhibitors of PKC or Src block leukocyte migration across endothelial cells [12], [24], [25]. In other reports, ICAM-1 activates extracellular signal-regulated kinase 1/2 (ERK1/2) and/or c-Jun N terminal kinase (JNK) [20], [26], [27]. However, the mechanisms for ICAM-1 activation of XO, PKC and ERK1/2 are not known. Moreover, the isoform of PKC in the ICAM-1 signaling pathway in endothelial cells is not known.

We recently reported that purified recombinant PKCα directly binds α-tocopherol and γ-tocopherol [28]. We also reported that α-tocopherol and γ-tocopherol function as a PKCα antagonist and agonist, respectively, during cofactor-dependent activation of purified recombinant PKCα or during oxidative activation of purified recombinant PKCα [28]. Furthermore, we reported that d-α-tocopherol inhibits VCAM-1-dependent oxidative activation of PKCα in mouse endothelial cells [6]. Whether ICAM-1 activation of PKC is regulated by tocopherols is not known, the isoform of PKC in ICAM-1 signaling in endothelial cells is not known, and whether ICAM-1 induces oxidative activation of PKC is not known. Therefore, to determine whether tocopherols regulate ICAM-1 signaling, we first determined determined the isoform of PKC activated by ICAM-1, we determined the mechanism for ICAM-1 activation of PKC and determined whether ICAM-1 stimulates oxidative activation of PKC. Then, we addressed our hypothesis that d-α-tocopherol and d-γ-tocopherol regulate ICAM-1 activation of PKC in human microvascular endothelial cells.

In this study, we demonstrated that ICAM-1 activated the PKC isoform PKCα and that this activation was not by direct oxidation of PKCα, although it was dependent on ICAM-1-induced XO-generated ROS in endothelial cells. ICAM-1-stimulated XO induced the activation of ERK1/2 that then activated PKCα. The ICAM-1-dependent activation of PKCα was inhibited by d-α-tocopherol but there was no effect of tocopherols on the upstream ERK1/2 activity. Furthermore, d-γ-tocopherol ablated d-α-tocopherol’s inhibition of ICAM-1-activated PKCα. This study demonstrates that d-α-tocopherol and d-γ-tocopherol have opposing regulatory functions for ICAM-1-activated PKCα in endothelial cells.

## Materials and Methods

### Cells

Human microvascular endothelial cells from the lung (HMVECLs) (CC-Lonza, Walkersville, MD) were grown in EGM-MV endothelial growth medium plus 5% FCS (CC-3125, Lonza) and were used at passage 2–6.

### Tocopherol Reagents

d-α-tocopherol >98% (MP Biomedicals) and d-γ-tocopherol (Supelco) 99.9% purity was confirmed by HPLC with electrochemical detection as described below.

### Reagents

For ICAM-1 crosslinking, we used mouse anti-human ICAM-1 (clone 84H10, catalog number MCA532) and goat anti-mouse IgG (catalog number STAR117); for ICAM-1 expression by flow cytometry we used mouse anti-human ICAM-1 conjugated FITC (clone 84H10, catalog number MCA532F) from AbD Serotec (Raleigh, NC). For crosslinking of PECAM-1, we used mouse anti-human CD31 (catalog number 55544) from BD PharMingen. Antibodies used for Western blots included rabbit anti-phospho-PKCα Thr^638^ (catalog number 9375), total PKCα (catalog number 2056), anti-phospho-p44/42 (ERK1/2) Thr^202^/Tyr^204^ (D13.14.14E)(catalog number 4370), rabbit p44/42 (ERK1/2) (137F5) (catalog number 4695), and anti-Rabbit IgG HRP-linked antibody (catalog number 7074) from Cell Signaling Technology, Danvers, MA. Rabbit anti-phospho-PKCβII Thr^641^ (catalog number 07–873) and anti-PKCβII rabbit monoclonal (catalog number 04–406) were from Millipore. Inhibitors Gö-6976 (catalog number G1171), allopurinol (catalog number A8003), U73122 hydrate (catalog number U6756), and PP2 (catalog number P0042) were obtained from Sigma-Aldrich. PD98059 (catalog number PHZ1164) and Ly294002 (catalog number PHZ1144) were from Invitrogen. Apocynin was from Acros Organics. ICAM-1 expression by the HMVECL’s was examined by immunolabeling and analysis with a BD Biosciences LSRII cytometer and FlowJo software.

### Cytotoxicity Assay

The Vybrant Cell Metabolic Assay Kit (V-23110, Invitrogen, MA, USA) was used to detect the metabolic activity of the HMVECLs treated with d-α-tocopherol, d-γ-tocopherol or pharmacological inhibitors at the doses indicated. In this assay, damaged release the cytosolic enzyme G6PD from damaged cells into the medium. G6PD reduces non-fluorescent resazurin (R-12204) to red-fluorescent resorufin. The resulting fluorescence is proportional to the amount of G6PD released into the medium, and this release correlates with the number of dead cells in the sample (Assay kit V-23111). Fluorescence was measured in a microplate reader (excitation/emission ∼530/590 nm). The % relative cytotoxicity was calculated as follows: 100 x (fluorescence of the experimental cells-background of untreated control cells)/(fluorescence of the fully lysed cells-background of the untreated control cells).

### Tocopherol Loading of Endothelial Cells

80% confluent monolayers of HMVECLs were incubated with 10 ng/ml TNFα for 6 hr. Then, d-α-tocopherol, d-γ-tocopherol, or the solvent control 0.01% dimethyl sulfoxide (DMSO) were added overnight. To determine the endothelial cell tocopherol levels, HMVECL cells were suspended and homogenized in absolute ethanol with 5% ascorbic acid on ice. Homogenates were extracted with an equal volume of 0.1% butylated hydroxytoluene in hexane to prevent oxidation and increase recovery of tocopherol. The samples were vortexed and then centrifuged for 5 minutes at 2000 rpm at room temperature. The hexane layer was removed to a separate vial and the hexane extraction step was repeated two more times for a total of three hexane extractions per sample. The hexane layer was dried under nitrogen and stored at −20°C. The samples were reconstituted in methanol and then tocopherols were separated using a reverse phase C18 HPLC column and HPLC (Waters Company, Milford, MA) with 99% methanol-1% water and 1% lithium perchlorate as a mobile phase. Tocol was used as internal standard. Tocopherols were detected with an electrochemical detector (ECD) (potential 0.7V) (Waters Company).

### Antibody-coated Beads

Streptavidin-coated 9.9 µm diameter beads (80 µl) (Bangs Laboratories) were labeled with 24 µg of biotin-conjugated goat anti-mouse Ig in 375 µl of PBS with gentle rocking for 1 hr at 4°C and then washed three times [29]. These beads were incubated with 16 µg of rat anti-human ICAM-1 or rat anti-human PECAM-1 in 375 µl of PBS with gentle rocking for 1 hr at 4°C and then washed.

### Western Blotting

Cell lysates were analyzed by 10% SDS-PAGE electrophoresis and transferred to nitrocellulose membranes using the semi-dry method according to manufacturer’s instructions (Bio-Rad). The membranes were blocked in 5% non-fat dried milk in Tris-buffered saline plus 0.1% Tween 20 (TBS-T) for 1 hour at room temperature and washed 3 times for 5 minutes in TBS-T. Membranes were incubated with primary antibodies in TBS-T plus 5% milk overnight, washed 3 times for 5 minutes in TBS-T, incubated with secondary antibodies in TBS-T plus 5% milk for 1 hour, washed 3 times for 10 minutes in TBS-T, and examined for detection with enhanced chemiluminescence (Amersham) and autoradiography. Densitometry was performed using Image J software (NIH). The data were presented as the fold increase in the ratio of relative intensity of the band/the relative intensity of band for the loading control (total PKCα, total PKCβII or total ERK1/2).

### Assessment of Cysteine Oxidation

Cysteine oxidation was assessed using N-(biotinoyl)-N’-(iodoacetyl)ethylenediamine (BIAM) according to the protocol previously described [30]. This is a sensitive method that detects proteins that contain H_2_O_2_-sensitive Cys residues [30]. Endothelial cells were stimulated with anti-ICAM-1-coated beads or anti-PECAM-1-coated beads. These cells were lysed in a buffer containing 50 mM 4-morpholine ethane sulfonic acid (MES), 100 mM NaCl, 50 µM phenylmethylsulfonyl fluoride, 1.0 µg/ml leupeptin, 1.0 µg/ml aprotinin, and 0.5% Triton X-100, 100 µM BIAM (pH 6.5) for 30 min [30]. The buffer was made fresh, rendered free of O_2_ by bubbling with N_2_ at a low flow rate for 1 hr. The positive control include lysates oxidized with 200 µM H_2_O_2_ for 20 min before addition of BIAM. The BIAM reaction was terminated by addition of 20 mM β-mercaptoethanol. PKCα was immunoprecipitated and separated by SDS-PAGE. Proteins labeled with BIAM were detected with HRP-conjugated streptavidin and ECL. BIAM reacts only with nonoxidized cysteines. Thus, loss of BIAM labeling indicates oxidation.

### Statistics

Data were analyzed by a one way ANOVA followed by Tukey’s multiple comparisons test (SigmaStat, Jandel Scientific, San Ramon, CA).

## Results

### ICAM-1 Crosslinking Activates PKCα but not in PKCβII in HMVECLs

**Figure 1:**
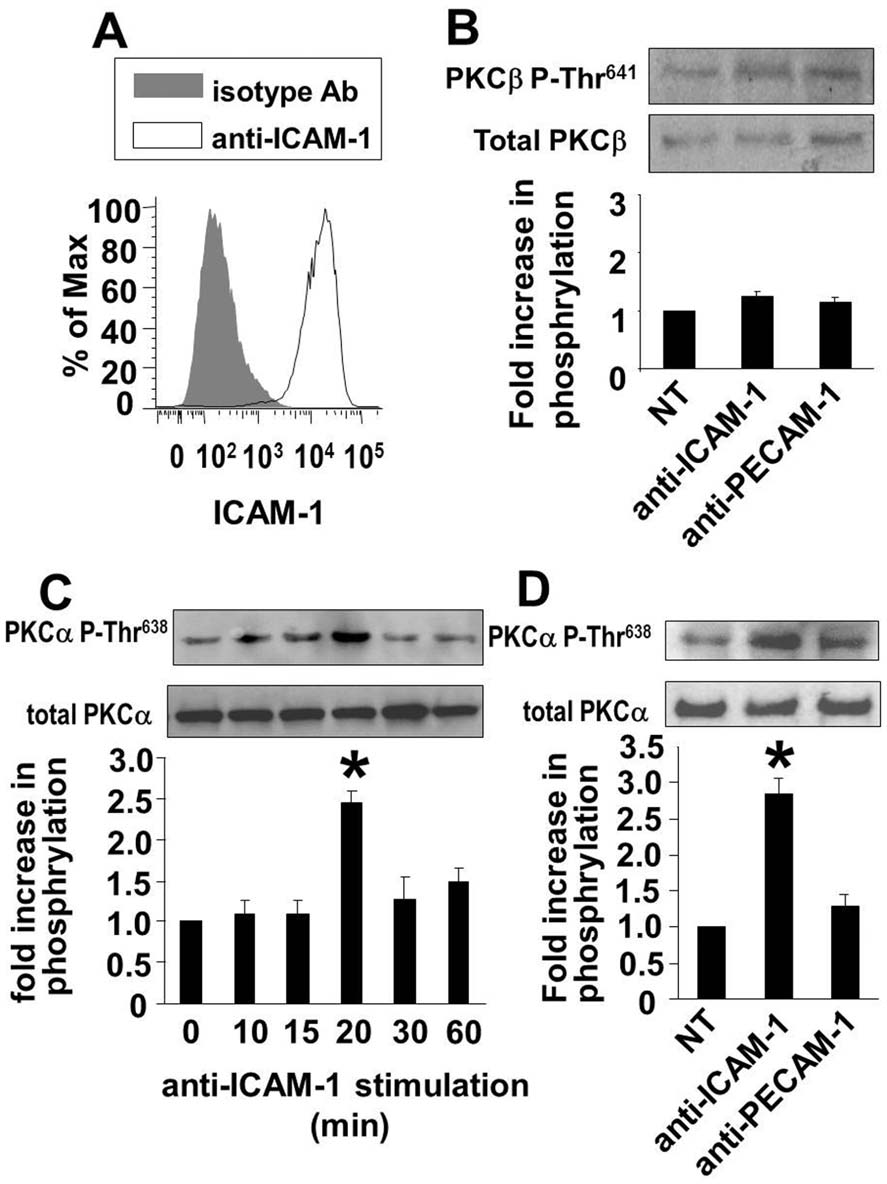
ICAM-1 activates PKCα but not PKCβII in HMVECLs. A ) At 70% confluence, HMVECLs cells pretreated with 10 ng/ml TNFα to induce ICAM-1 expression. At 24 hrs, the cells were suspended and immunolabeled with anti-ICAM-1 antibodies and examined by flow cytometry for ICAM-1 expression. **B–D**) HMVECLs were pretreated with TNFα as in panel A. At 24 hrs, the endothelial cells were nonstimulated (NS) or stimulated with a confluent monolayer of anti-ICAM-1-coated beads or the control anti-PECAM-1-coated beads for 20 minutes in **B** and **D** or for the times indicated in **C**. The cells were lysed and the activation of PKCβII and PKCα was examined by western blot with **B**) anti-phospho-PKCβII^Thr641^ or anti-PKCβII or **C–D**) anti-phospho- PKCα^Thr638^ or anti-PKCα. Shown are representative blots. Shown are the mean ± SEM of 3 experiments. NT, nontreated. *, p<0.05 as compared to the nontreated (NT) groups.

It is reported that ICAM-1 activates endothelial cell PKC [24] but the PKC isoform is not known. Therefore, we determined whether ICAM-1 activates PKCα or PKCβ. For these studies, primary cultures of human microvascular endothelial cells from the lung (HMVECLs) were stimulated overnight with 10 ng/ml TNFα to induce ICAM-1 expression. ICAM-1 was expressed as determined by immunofluorescence labeling and flow cytometry **(** **Figure 1A** **)**. Then, the TNFα-pretreated HMVECLs were activated with anti-human ICAM-1-coated beads for 10–60 min and examined for autophosphorylation at PKCαThr^638^. Phosphorylated PKCαThr^638^is the active form of PKCα and is required for enzyme activity [31], [32]. At 20 min, ICAM-1 crosslinking induced an increase in phosphorylation of PKCαThr^638^ (**Fig.** **1C**). In contrast to ICAM-1 activation of PKCα, PKCβII was not activated by anti-ICAM-1-coated beads as indicated by no increase in phosphorylation of PKCβII Thr^641^ (**Fig. 1B**). Anti-PECAM-1-coated control beads did not activate phosphorylation of PKCαThr^638^ or PKCβII Thr^641^ (**Fig. 1B,D**). These data indicate that ICAM-1 activates the PKCα isoform.

### ICAM-1 Activation of PKCα Phosphorylation in HMVECLs is Mediated by a Xanthine Oxidase Pathway

**Figure 2:**
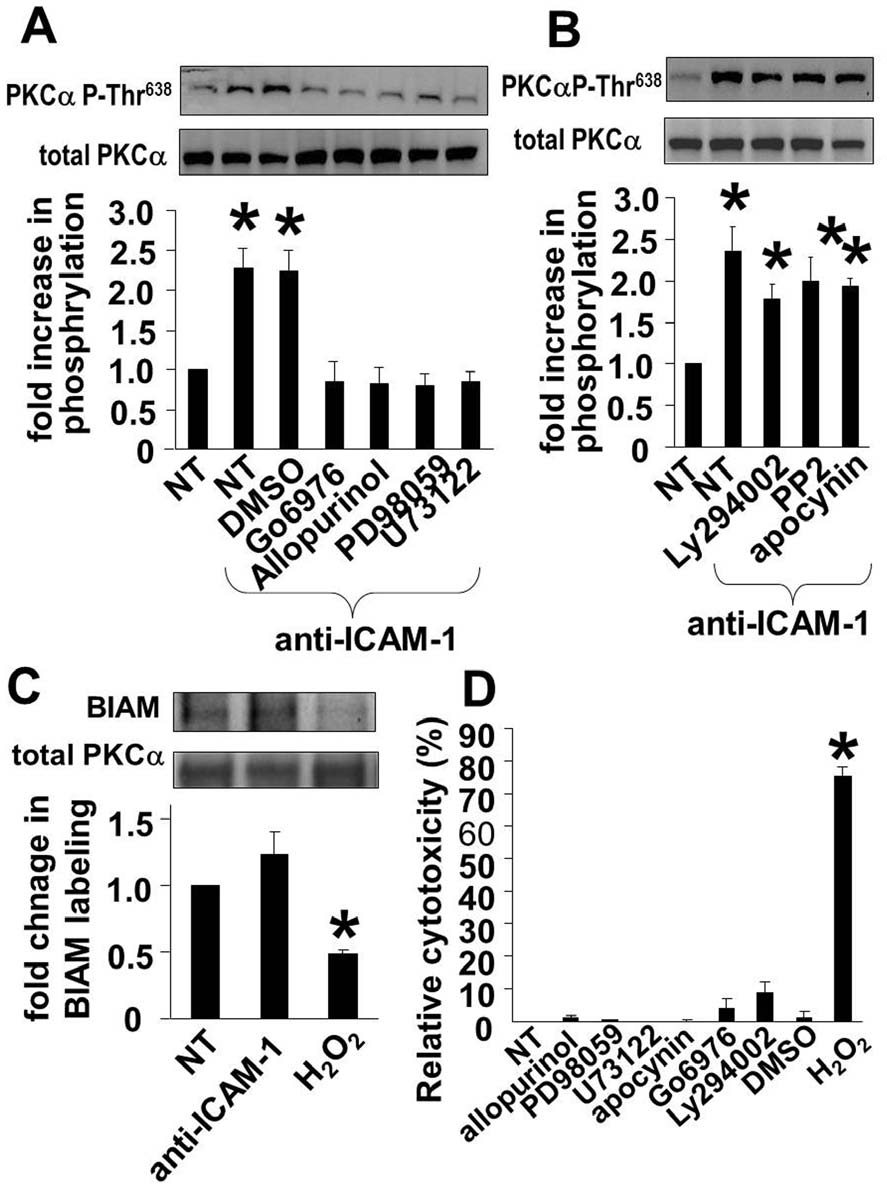
Anti-ICAM-1 stimulation induces an increase in PKCα^Thr 638^ phosphorylation through XO/PLC/ERK1/2 activities but not by oxidation. A,B,D ) 70% confluent monolayers of HMVECLs were pretreated with TNFα to induce ICAM-1 expression. Then, the endothelial cells were were treated for 1 hr with the pharmacological inhibitors allopurinol (0.3 mg/ml), PD98056 (20 µM), U73122 (10 µM), apocynin (4 mM), Ly294002 (100 nM), Go6976 (2.3 nM), PP2 (10 µM), apocynin (4 mM) or the solvent control DMSO (0.01%). The cells were stimulated with anti-ICAM-1-coated beads and examined for (**A,B**) PKCα^Thr638^ phosphorylation and total PKCα by western blot or examined for (**D**) cytotoxicity by the Vybrant cytotoxicity assay. In panel D, 200 µM H_2_O_2_ was used as a positive control for cytotoxicity. **C**) To label non-oxidized cysteines, BIAM was added to the cell lysates. PKCα was immunoprecipitated and BIAM labeling was detected by western blot with HRP-conjugated streptavidin. The blots were reprobed for total PKCα. The positive control for oxidation includes lysates oxidized with 200 µM H_2_O_2_ for 20 min before addition of BIAM. Loss of BIAM labeling in the western blot indicates PKCα oxidation. Shown are the means ± SEM from 3 experiments. *, p<0.05 compared to nonstimulated (NS) controls.

In endothelial cells, ICAM-1 ligation activates xanthine oxidase (but not nitric oxide synthase) for the production of ROS [18], [19], [21], [33], [34]. ICAM-1 binding also induces a calcium/PLCγ_1_/PKC pathway for the activation of Src phosphorylation of cytoskeletal proteins during leukocyte diapedesis [24]. It is also reported that, in other signaling pathways, PI3 kinase can function in the activation of PKCα [35], [36], [37]. However, whether XO, PLC/PKC, Src, and PI3 kinase function in a single pathway during ICAM-1 activation of PKCα is not known. Therefore, we first determined whether XO, PLC, ERK, PI3 kinase or Src function in ICAM-1 activation of PKCα. For these studies, TNFα-stimulated HMVECLs were treated for 1 hr with inhibitors of XO, PLC, ERK, PI3 kinase, Src, or the solvent control 0.01% DMSO and then examined for ICAM-1 activation of PKCα. Inhibitor treatment of the HMVECLs was not cytotoxic as compared to the positive control 200 µM H_2_O_2_ **(** **Figure 2D** **)** and the inhibitors were used at doses that we and others have reported for studies with endothelial cells [29], [38], [39], [40], [41], [42], [43], [44]. The solvent control 0.01% DMSO did not affect ICAM-1 activation of PKCα **(** **Figure 2A** **)**. The PKCα inhibitor Go6876, which was used as a control for inhibition of PKCα autophosphorylation, inhibited ICAM-1-stimulated phosphorylation of PKCαThr^638^ **(** **Figure 2A** **)**. The Src kinase inhibitor PP2 (10 µM) and the PI3 kinase inhibitor Ly294002 (10 µM) did not block ICAM-1-induced activation of PKCα Thr^638^ (**Fig. 2B**). In contrast, ICAM-1-induced activation of PKCα Thr^638^ was blocked by the PLC inhibitor U73122 (10 µM), ERK1/2 inhibitor PD98059 (10 µM), and xanthine oxidase inhibitor allopurinol (0.3 mg/ml) (**Fig. 2A**), suggesting that PLC, ERK1/2 and XO-generated ROS are required for ICAM-1 activation of PKCα.

### PKCα is not Oxidized during its Activation by ICAM-1

XO-generated ROS were required for ICAM-1 activation of PKCα in endothelial cells (**Figure 2A**), but it is not known whether PKCα is activated by oxidation. Oxidative activation of PKCα has been reported for VCAM-1 signaling through NADPH oxidase-generated ROS [44]. Therefore, we determined whether ICAM-1 stimulates oxidation of PKCα and whether NADPH oxidase has a role in ICAM-1 activation of PKCα. Treatment of TNFα-stimulated HMVECLs for 1 hr with the NADPH oxidase inhibitor apocynin (4 mM) did not block ICAM-1 activation of PKCα **(** **Figure 2B** **)**. To examine oxidation of PKCα, TNFα-stimulated HMVECLs were stimulated with anti-ICAM-1 and then lysed in the presence of BIAM which binds to non-oxidized cysteines. ICAM-1 was immunoprecipitated and BIAM labeling was detected with HRP-conjugated streptavidin in a western blot. Loss of BIAM labeling indicates cysteine oxidation of PKCα. Anti-ICAM-1-coated beads did not induce oxidation of PKCα cysteines, compared to the positive control, lysates treated with 200 µM H_2_O_2_ (**Fig. 2C**). Therefore, the ROS that are generated by xanthine oxidase during ICAM-1 activation of PKCα do not directly oxidize and activate PKCα.

### ICAM-1 Activation of PKCα Phosphorylation in HMVECLs is Mediated by Xanthine Oxidase Stimulation of ERK1/2

**Figure 3:**
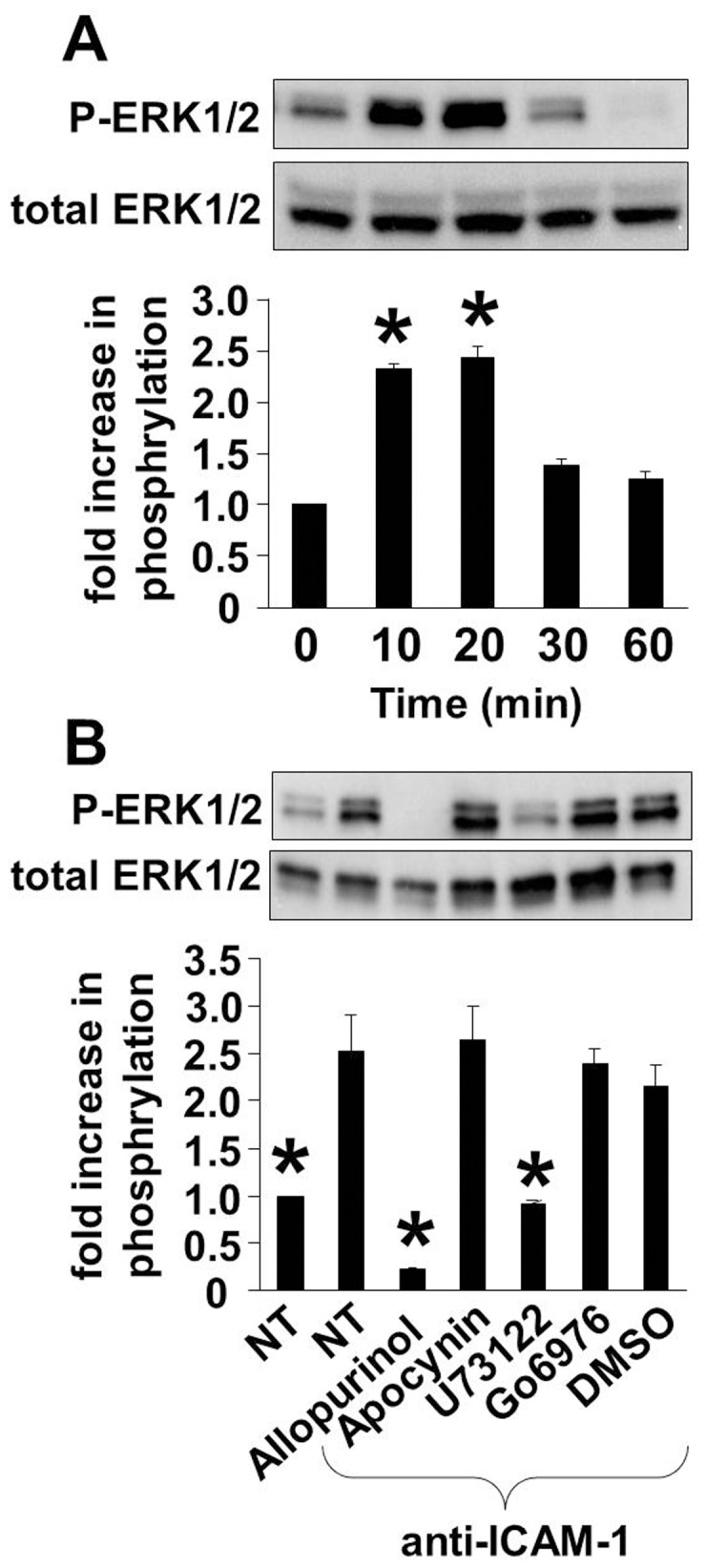
ICAM-1 activation of PKCα phosphorylation in HMVECLs is mediated by xanthine oxidase stimulation of ERK1/2. 70% confluent monolayers of HMVECLs were pretreated with TNFα to induce ICAM-1 expression. **A**) Time course for anti-ICAM-1-coated bead stimulation of ERK1/2 Thr^202^/Tyr^204^ phosphorylation (P-ERK1/2). *, p<0.05 as compared to the nontreated control group. **B**) TNFα-pretreated HMVECLs were incubated for 1 hr with the pharmacological inhibitors allopurinol (0.3 mg/ml), U73122 (10 µM), and Go6976 (2.3 nM), apocynin (4 mM), or the solvent control DMSO (0.01%). The cells were nonstimulated (NS) or stimulated with anti-ICAM-1-coated beads and examined by western blot for ERK1/2 Thr^202^/Tyr^204^ phosphorylation and total ERK1/2. Shown are the means ± SEM from 3 experiments. Panel A: *, p<0.05 as compared to 0 minutes. Panel B: *, p<0.05 as compared to the anti-ICAM-1-stimulated group.

It is reported that ICAM-1 binding induces activation of extracellular signal-regulated kinase 1/2 (ERK1/2) [20], [26], [27]. In addition, ICAM-1-dependent ROS production is reported to activate mitogen-activated protein (MAP) kinases [3], [6]–[9]. Therefore, since ICAM-induced ROS did not oxidize PKCα (**Figure 2C**) and it is reported that ICAM-1 activates ERK1/2, we determined whether xanthine oxidase activates ERK1/2 that then activates PKCα. We determined that ICAM-1 activates phosphorylation of ERK1/2 Thr^202^/Tyr^204^ in TNFα-treated HMVECLs at 20 minutes. (**Figure 3A**). To determine whether ICAM-1-induced ERK1/2 activity requires XO, PLC or PKCα activation, TNFα-activated cells were treated with 0.3 mg/ml allopurinol (XO inhibitor), 10 µM U73122 (PLC inhibitor) or 2.3 nM Gö6976 (PKCα inhibitor) (**Fig. 3B**). Pretreatment of HMVECLs with allopurinol and U73122 inhibited ICAM-stimulated phosphorylation of ERK1/2 Thr^202^/Tyr^204^ **(** **Figure 3B** **)** and PKCα **(** **Figure 2A** **)**. In contrast, ICAM-1-activated ERK1/2 was not blocked by the PKCα inhibitor Gö6976 **(** **Figure 3B** **),** indicating that PKCα is not upstream of ERK1/2 in ICAM-1 signaling. These data suggest that ICAM-1 induces activation of XO that activates ERK1/2 and this then activates PKCα.

### Treatment of HMVECLs with Isoforms of the Antioxidant Vitamin E

**Figure 4:**
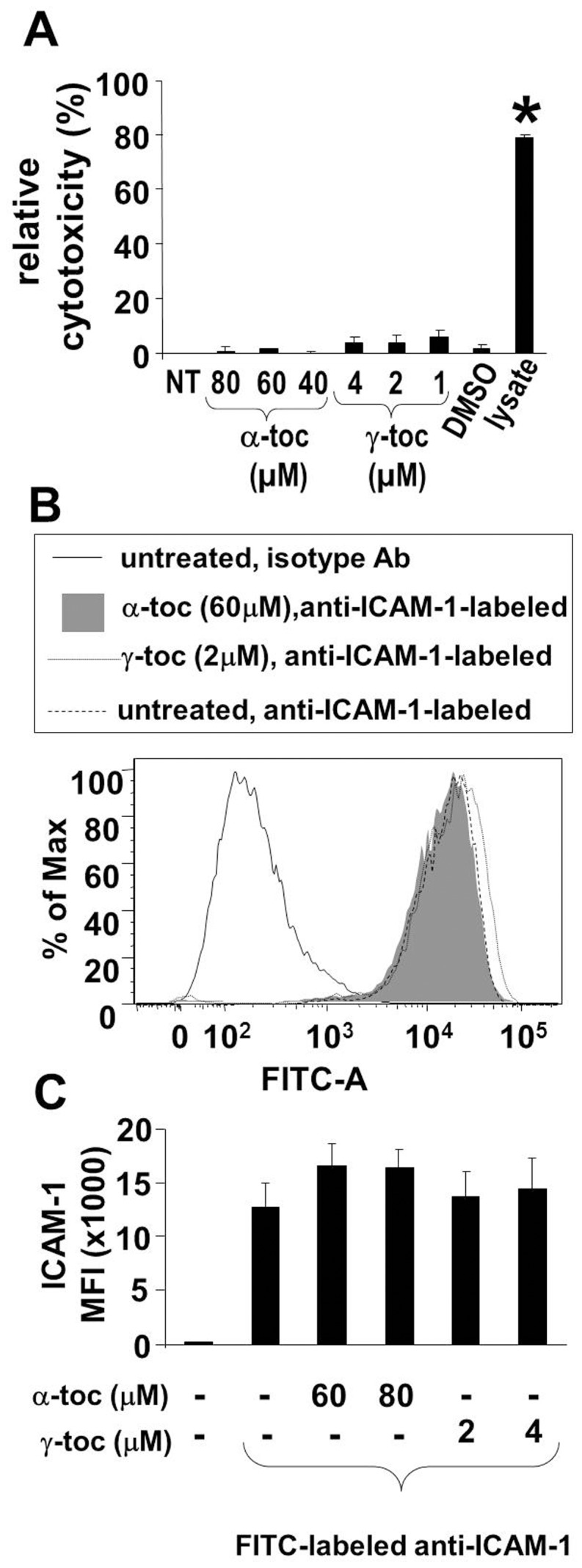
Tocopherol treatment of TNFα-stimulated HMVECLs was not cytotoxic and did not alter ICAM-1 expression. 70% confluent monolayers of HMVECLs were pretreated for 6 hrs with TNFα (10 ng/ml) to induce ICAM-1 expression. Then, the endothelial cells were treated for 16 hrs with α-tocopherol (α-toc) (40, 60 or 80 µM), γ-tocopherol (γ-toc) (1, 2 or 4 µM) or the vehicle control DMSO (0.01%). The cells were examined for cytotoxicity with the **A**) Vybrant Cytotoxicity Assay or examined by **B,C**) immunolabeling with FITC-labeled anti-ICAM-1 and flow cytometry. Shown are the means ± SEM from 3 experiments. MFI, mean fluorescence intensity. *, p<0.05 compared with non-treated (NT) cells.

We previously reported a) that ICAM-1-dependent and VCAM-1-dependent recruitment of leukocytes in vivo is regulated by tocopherols, b) that in microvascular endothelial cells, d-α-tocopherol inhibits VCAM-1 oxidative activation of PKCα, and c) that d-γ-tocopherol ablates this inhibition by d-α-tocopherol [6]. This tocopherol regulation of VCAM-1 signaling occurred without altering VCAM-1 expression [6]. We have also reported that these tocopherol isoforms can directly bind to and regulate purified recombinant PKCα activity [28]. However, it is not known whether tocopherols regulate ICAM-1 activation of PKC. To examine tocopherol function during ICAM-1 signaling, HMVECLs were pretreated with TNFα for 6 hours to induce ICAM-1 expression and then the cells were incubated overnight with d-α-tocopherol or d-γ-tocopherol to generate tocopherol levels in the HMVECLs equivalent to the levels of tocopherol in lung tissue. Tocopherol levels were determined by HPLC with ECD [6]. Treatment with 40–80 µM d-α-tocopherol or 1–4 µM d-γ-tocopherol was not toxic to the cells as determined by the Vybrant Cell Metabolic Assay **(** **Figure 4A** **).** The tocopherols did not affect ICAM-1 expression by the HMVECL’s as determined by immunolabeling and flow cytometry **(** **Figure 4B,C** **)**. Treatment of HMVECLs with 60 µM α-tocopherol or 2 µM γ-tocopherol resulted in 10 µg α-tocopherol/g cells and 2.8 µg γ-tocopherol/g cells (**Table 1**). This is consistent with reports that human and mouse lung tissue levels of α-tocopherol are 9–10 µg/g of tissue [45] and that γ-tocopherol tissue levels are 5–10 fold lower than α-tocopherol [6]. In vivo, γ-tocopherol tissue levels are lower because of the preferential transfer in the liver by α-tocopherol transfer protein [46]. In human plasma, the normal range of d-α-tocopherol is 20–30 µM, whereas 60 µM d-α-tocopherol is achieved in plasma by oral supplementation with 200–800 IU of d-α-tocopherol per day [**47**]. Therefore, HMVECLs were loaded in vitro with tocopherols at the levels of tocopherols reported for lung tissue.

**Table 1:** Uptake of tocopherols by HMVECLs.

Treatment	µg tocopherol/g cells	
	α-toc	γ-toc
**DMSO (0.01%)**	**0.28±0.03**	**0.13±0.01**
**α-toc (80 µM)**	**12.61±1.36** *	**0.35±0.14**
**α-toc (60 µM)**	**10.08±1.07** *	**0.15±0.02**
**α-toc (40 µM)**	**5.4±.4** *	**0.15±0.02**
**γ-toc (5 µM)**	**0.86±0.13**	**8.84±0.42** *
**γ-toc (4 µM)**	**0**	**3.76±0.37** *
**γ-toc (2 µM)**	**0**	**2.85±0.38** *
**γ-toc (1 µM)**	**0**	**0.55±0.07** *
**α-toc (80 µM) + γ-toc (2 µM)**	**10.7±2.1** *	**2.5±0.14** *
**α-toc (60 µM) + γ-toc (2 µM)**	**9.5±1.8** *	**2.7±0.4** *

At 70% confluence, HMVECLs cells were stimulated for 6 hrs with 10 ng/ml TNFα and then were treated for 16 hrs with a dose curve of α-tocopherol (α-toc) and/or γ-tocopherol (γ-toc) or with the vehicle control (DMSO 0.01%). Cells were washed, cell pellet weighed and tocopherol uptake was measured by HPLC/ECD. Data are expressed as µg tocopherol per g cells. Shown is the mean± SEM n = 3–5.

* p<0.05 compared to the DMSO control.

### D-α-tocopherol Inhibits ICAM-1-stimulated Activation of PKCα but not ERK1/2 in HMVECLs; the Inhibition by d-α-tocopherol is Abrogated by d-γ-tocopherol

**Figure 5:**
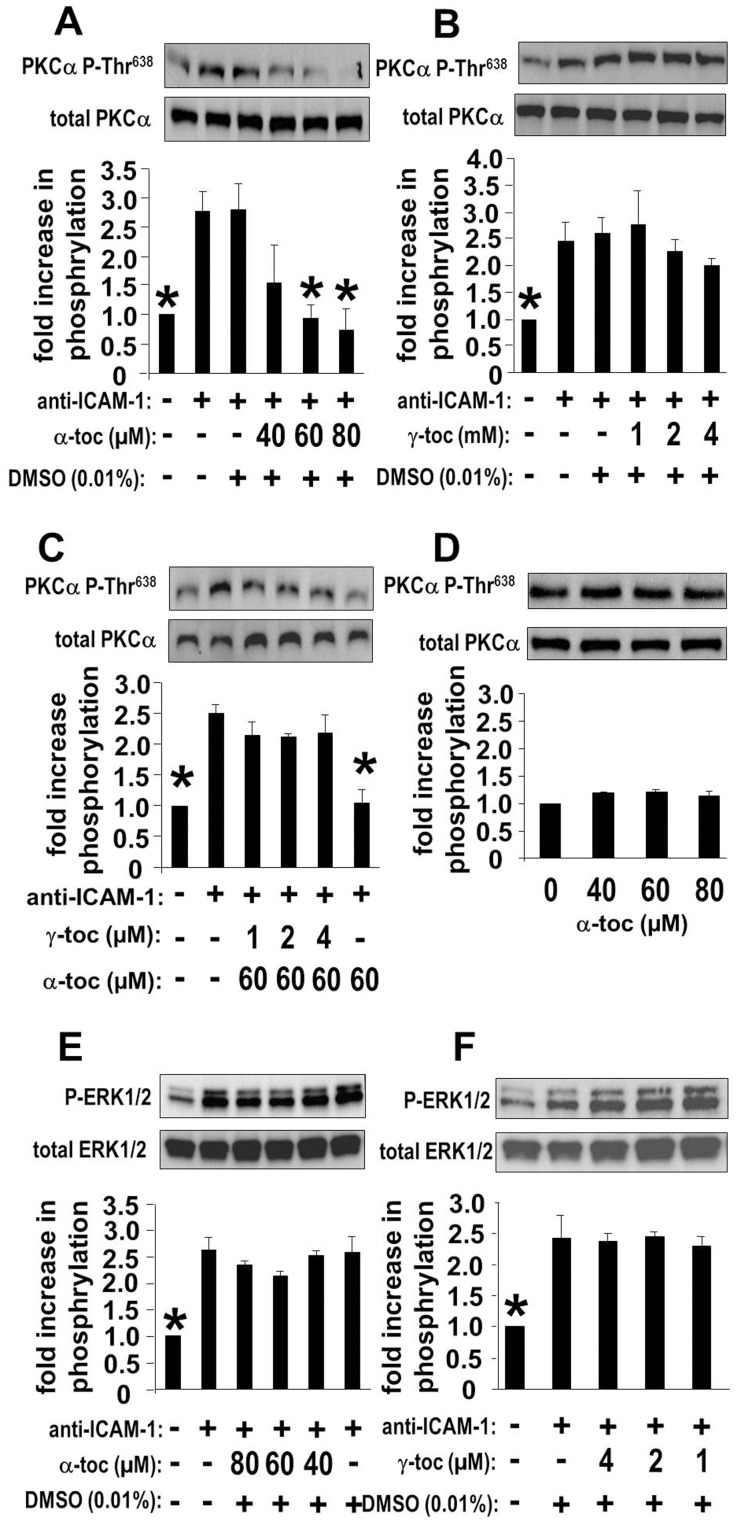
D-α-tocopherol inhibits ICAM-1-activated PKCα but not ERK1/2 in HMVECLs and the inhibition by d-α-tocopherol is abrogated by d-γ-tocopherol. At 70% confluence, HMVECLs cells were pretreated with TNFα for 6hrs and then treated with tocopherols or the solvent control 0.01% DMSO for 16 hrs. **A**) d-α-tocopherol (α-toc) regulation of ICAM-1-stimulated PKCα Thr^638^ phosphorylation (PKCα P-Thr^638^). **B**) d-γ-tocopherol (γ-toc) regulation of ICAM-1-stimulated PKCα P-Thr^638^. **C**) d-α-tocopherol + d-γ-tocopherol regulation of ICAM-1-stimulated PKCα P-Thr^638^. **D**) d-α-tocopherol does not alter background PKCα P-Thr^638^ (no anti-ICAM-1). **E**) d-α-tocopherol does not regulate ERK1/2 Thr^202^/Tyr^204^ phosphorylation (P-ERK1/2). **F**) d-γ-tocopherol does not regulate ERK1/2 Thr^202^/Tyr^204^ phosphorylation. Shown are the means ± SEM from 3 experiments. *, p<0.05 as compared with the anti-ICAM-1-stimulated groups.

We determined whether ICAM-1 activation of PKCα was inhibited by preloading tocopherols in HMVECLs as in TABLE 1. Treatment with the tocopherols did not alter total PKCα expression **(** **Figure 5A–D** **)**. ICAM-1 activation of PKCα Thr^638^ phosphorylation was inhibited by d-α-tocopherol (**Figure 5A**) but not by d-γ-tocopherol (**Figure 5B**). Interestingly, d-γ-tocopherol ablated d-α-tocopherol’s inhibition of ICAM-1-activated PKCα **(** **Figure 5C** **)**. Basal levels of PKCα Thr^638^ phosphorylation were not affected by d-α-tocopherol (**Figure 5D**) or d-γ-tocopherol (data not shown). In contrast to the tocopherol regulation of ICAM-1 activation of PKCα, these tocopherols did not affect ICAM-1 activation of ERK1/2 **(** **Figure 5E,F** **)**. These data suggest that during ICAM-1 signaling, the tocopherols do not function to block xanthine oxidase-mediated activation of ERK1/2. The tocopherols function, at least, downstream of ERK1/2 to regulate PKCα activity.

## Discussion

In this report, we identify a mechanism for ICAM-1 activation of PKC in endothelial cells and determined that this activation of PKC is regulated by tocopherols. We determined that stimulation of ICAM-1 activated PKCα but not PKCβ. Antibody crosslinking of ICAM-1 activated XO, PLC, and ERK1/2 which then activated PKCα. ICAM-1 activation of PKCα was blocked by the PLC inhibitor U73122, ERK1/2 inhibitor PD98059, and xanthine oxidase inhibitor allopurinol. ERK1/2 activation was blocked by inhibition of XO and PLC but not by inhibition of PKCα, indicating that ERK1/2 is downstream of XO and upstream of PKCα during ICAM-1 signaling. ICAM-1 activation of PKCα was not blocked by inhibitors of Src or PI3 kinase. During ICAM-1 activation of PKCα, the XO-generated ROS did not oxidize PKCα. Instead, crosslinking ICAM-1 stimulated XO/ROS which activated ERK1/2 that then activated PKCα. The ICAM-1 activation of PKCα was inhibited by α-tocopherol treatment of the endothelial cells and this inhibition by α-tocopherol was abrogated by γ-tocopherol. In contrast, the tocopherols did not alter the ICAM-1 activation of PKCα’s upstream signal, ERK1/2. This suggests that since ERK1/2 activation was not affected by tocopherols but ERK1/2 activation was dependent on XO, the antioxidant properties of the tocopherols did not function to block the XO-induced ERK1/2 that leads to activation of PKCα.

Separate reports indicate that ICAM-1 can activate PKC, XO, PLC or ERK1/2 [20], [26], [27], but the mechanisms for activation of these signals in endothelial cells was not known. In brain endothelial cell lines, ICAM-1 binding induces a calcium/PLCγ_1_/PKC pathway [24]. They also report that pharmacological inhibition of PKC blocks transendothelial migration [24]. Chelating calcium or inhibitors of PKC or Src block leukocyte migration across endothelial cells [12], [24], [25]. However, the PKC isoform activated by ICAM-1 signaling was not known. Furthermore, the mechanism for ICAM-1 activation of PKC was not known. We report here that crosslinking ICAM-1 on human microvascular lung endothelial cells activates endothelial cell PKCα but not PKCβ. In other signaling pathways, PKC can function upstream of ROS production in that PKC can activate NADPH oxidase for the release of ROS in neutrophils and monocytes [48], [49]. During VCAM-1 signaling, NADPH oxidase-generated ROS directly oxidize and activate PKCα followed by downstream activation of ERK1/2 [50]. In contrast to VCAM-1-induced oxidation of PKCα [50], we demonstrated that ICAM-1 signals do not induce oxidation of PKCα. Instead, ICAM-1-stimulated XO/ROS induce activation of ERK1/2 which then activates PKCα. Therefore, the mechanism for ICAM-1 activation of PKCα is different than the mechanism for VCAM-1 activation of PKCα.

We have previously reported that the *in vivo* ICAM-1-dependent and VCAM-1-dependent recruitment of lymphocytes and eosinophils in allergic lung inflammation is inhibited by d-α-tocopherol and elevated by d-γ-tocopherol [6]. Furthermore, VCAM-1 induces oxidative activation of endothelial cell PKCα which is blocked by treatment with d-α-tocopherol [44]. This d-α-tocopherol inhibition of VCAM-1-stimulated oxidative activation of PKCα is ablated by d-γ-tocopherol [44]. Although the mechanism for VCAM-1 activation of PKCα is through oxidation of PKCα whereas we report here that ICAM-1 activation of PKCα is not through oxidation of PKCα, we determined that treatment of endothelial cells with d-α-tocopherol inhibits ICAM-1 activation of PKCα and d-γ-tocopherol ablates the inhibition by d-α-tocopherol.

We have recently reported that d-α-tocopherol and d-γ-tocopherol can directly bind recombinant PKCα and regulate recombinant PKCα activity [28]. D-α-tocopherol and d-γ-tocopherol bind to the regulatory C1A domain of recombinant PKCα. Recombinant PKCα activity is decreased by d-α-tocopherol and increased by d-γ-tocopherol [28]. Furthermore, d-α-tocopherol inhibition of recombinant PKCα is blocked by d-γ-tocopherol [28]. However, these studies were with recombinant PKCα. It was not known whether tocopherols regulate ICAM-1 activation of PKC in endothelial cells. In the present study, ICAM-1-stimulated PKCα was inhibited by d-α-tocopherol and this inhibition by d-α-tocopherol was blocked by d-γ-tocopherol. This tocopherol regulation of PKCα occurred without tocopherol regulation of the upstream XO/ROS activation of ERK1/2. Thus, the tocopherol regulation of ICAM-1 activation of PKCα in endothelial cells is, at least, consistent with tocopherol interactions with PKCα.

There are conflicting reports with regards to whether tocopherols alter adhesion molecule expression. It is reported that in vitro α-tocopherol inhibits cytokine or oxidized LDL-induced expression of ICAM-1, VCAM-1, or E-selectin [51], [52], [53], [54], but whether in these studies the in vitro doses of tocopherols in cells were at concentrations of tocopherols found in tissues is not known. In contrast, in vivo d-α-tocopherol and d-γ-tocopherol do not alter VCAM-1 expression and, when tocopherols are loaded in endothelial cells in vitro at concentrations found in vivo, tocopherols do not alter VCAM-1 expression or lymphocyte binding to endothelial cells in vitro [6]. In our current report, HMVECL’s were stimulated with TNFα for 6 hours to induce ICAM-1 expression and then supplemented overnight with doses of tocopherols to generate cellular concentrations of tocopherols equivalent to that reported for lung tissue levels of tocopherols [6], [45]; there were no effects of tocopherols on ICAM-1 expression by the HMVECL’s in vitro. Therefore, without altering ICAM-1 expression, tocopherols regulated ICAM-1 activation of PKCα.

In reports of receptors other than ICAM-1, activation of PKC is inhibited by d-α-tocopherol treatment of muscle cells [55], [56], [57], [58], monocytes [59], [60], epithelial cells [61], endothelial cells [6], [48], [59], [62], and platelets [63]; in these studies, regulation by γ-tocopherol was not examined. In addition, although these reports indicate that α-tocopherol inhibits activation of PKC in cells, the mechanisms for the α-tocopherol inhibition of PKC activation are often not described. It is reported that in smooth muscle cells, d-α-tocopherol inhibits PKCα without inhibition of PKC expression [64]. Consistent with this, we report that d-α-tocopherol and d-γ-tocopherol did not alter total PKCα expression in endothelial cells. It is also reported that d-α-tocopherol activates smooth muscle PP2 and that purified PP2 can dephosphorylate both PKC and ERK1/2 [65], [66]. In our studies, d-α-tocopherol regulated PKCα without altering ERK1/2 activation during ICAM-1 signaling.

In summary, ICAM-1 activated XO, PLC, and ERK1/2 which then activated PKCα. Furthermore, d-α-tocopherol inhibited ICAM-1 activation of PKCα in human lung microvascular endothelial cells, without interfering with the upstream ICAM-1 stimulation of ERK1/2. The d-α-tocopherol inhibition of ICAM-1 activation of PKCα was blocked by d-γ-tocopherol. The tocopherol regulation of ICAM-1 activation of PKCα is consistent with a PKCα antagonist function of d-α-tocopherol and a PKCα agonist function of d-γ-tocopherol. Thus, we identified for the first time a mechanism for ICAM-1 activation of PKC in endothelial cells and determined that d-α-tocopherol and d-γ-tocopherol have opposing functions in regulating ICAM-1-activated PKCα in endothelial cells.
